# Seroprevalence of SARS-CoV-2 in Bhubaneswar, India: findings from three rounds of community surveys

**DOI:** 10.1017/S0950268821000972

**Published:** 2021-04-27

**Authors:** Jaya Singh Kshatri, Debdutta Bhattacharya, Ira Praharaj, Asit Mansingh, Debaprasad Parai, Srikanta Kanungo, Subrata Kumar Palo, Sidhartha Giri, Matrujyoti Pattnaik, Shakti Ranjan Barik, Girish Chandra Dash, Hari Ram Choudhary, Jyotirmayee Turuk, Nitya Nanda Mandal, Sanghamitra Pati

**Affiliations:** Indian Council of Medical Research-Regional Medical Research Centre, Nalco Square, Bhubaneswar, Odisha 751023, India

**Keywords:** COVID-19, infectious disease epidemiology, SARS-CoV-2, serosurvey

## Abstract

The study aims to estimate and compare the severe acute respiratory syndrome coronavirus 2 (SARS-CoV-2) seroprevalence, the fraction of asymptomatic or subclinical infections in the population, determine the demographic risk factors and analyse the antibody development at different time points among adults in Bhubaneswar city, India. This was a serial three-round cross-sectional, community-based study where participants were selected from the residents of Bhubaneswar city using multi-stage random sampling. Blood samples were collected during household visits along with demographic and clinical data from every participant. Total anti-SARS-CoV-2 antibody present in serum was assessed using the electro-chemiluminescence immunoassay platform. Temporal comparisons of the community seroprevalence were performed against the detected number of cumulative cases, active cases, recoveries and deaths. A total of 3693 participants were enrolled in this study with a cumulative non-response rate of 18.33% in all the three rounds. The gender-weighted seroprevalence for the city in the first round was 1.55% (95% confidence interval (CI) 0.84–2.58), second round was 5.27% (95% CI 4.13–6.59) and in the third round was 49.04% (95% CI 46.39–51.68). In the first round, the seroprevalence was found to be highest in the elderly population, whereas the seroprevalence for the second and third phases was highest in the age group of 30–39 years. Seroprevalence showed an increasing trend over the three time periods, with the highest seropositivity rates among individuals sampled between 16 and 18 September 2020. By the third round, 93.93% of those who had previously been tested positive by real-time reverse transcription polymerase chain reaction had seroconversion and 46.57% of those who had been tested negative also showed seroconversion. Infection to case ratio during first round was 27.05, for second round and third round it was 5.62 and 17.91, respectively.

## Introduction

The coronavirus disease 2019 (COVID-19) pandemic caused by severe acute respiratory syndrome- coronavirus 2 (SARS-CoV-2) has affected 219 countries worldwide, and as of 24th February 2021, 111.59 million confirmed cases were reported globally, with more than 2.47 million deaths [1]. In India, the first COVID-19 case was notified on 30th January 2020. By 24th February 2021, the country had, according to official statistics, over 11.031 million confirmed cases and over 156 567 deaths [2].

India remains the second country after USA which is most severely impacted by the COVID-19 pandemic. Infections with SARS-CoV-2 may have clinical manifestations which can be symptomatic (mild to severe), or asymptomatic. However, only a proportion of all COVID-19 cases are identified through routine epidemiological surveillance, which is done mostly by contact tracing. A population-based seroepidemiological study can measure the extent of the population that has antibodies against SARS-CoV-2. Such studies can estimate the proportion of the population exposed to the virus and the proportion of the population that remains susceptible to the virus. The antibodies developed in the population can act as a marker of total or partial immunity. Probability sampling from a target population can give true representativeness and can help in providing more evidence-based public health strategies. Findings of first nationwide population-based serological survey conducted by Indian council of Medical Research (ICMR), New Delhi, reveals that 0.73% of adults (>18 years) in India were exposed to SARS-CoV-2 infection, amounting for 6.4 million cases in total by early May 2020 [3].

Existing data from animal studies with other coronaviruses and whatever follow-up data are available during the ongoing COVID-19 pandemic suggest that SARS-CoV-2 infection provides some extent of immunity [4]. Although nucleic acid detection-based or antigen-detection-based tests are useful for detecting and tracking active cases of COVID-19, given that asymptomatic infection also constitutes a considerable proportion of SARS-CoV-2 positives, to infer the true extent of infection in the community, it is important to use the information provided by serological tests as well. Serosurveillance studies carried out at different points of time in a pandemic can help monitor the spread of infection in a community and region. Knowing the true infection rates are important when calculating the infection fatality rate (IFR), considered as a more appropriate parameter compared to case fatality rate (CFR) while assessing a novel and newly emerged pathogen such as SARS-CoV-2 [5–7].

Apart from revealing the community level immunity, and estimating contributions from asymptomatic, presymptomatic or subclinical infections, serosurveillance for an emerging infectious pathogen such as SARS-CoV-2 can help elucidate antibody kinetics at the population level.

Our study provides an overall picture of repeated serological surveys on anti-SARS-CoV-2 antibodies in the city of Bhubaneswar, India. The study aims to estimate and compare the seroprevalence, the fraction of asymptomatic or subclinical infections in the population, determine the demographic risk factors and analyse the evolution of the antibody development associated with SARS-CoV-2 infection at different time points among adults in Bhubaneswar city, and ascertain the association with the progression of the pandemic.

## Methodology

This was a population-based serial cross-sectional study (three rounds), conducted in the city of Bhubaneswar, the capital and largest city of the state of Odisha, India, with a population of about 1 million. The three rounds were carried out in the months of July, August and September 2020.

### Sampling design and sample size

The study population was randomly selected from the community members of the municipal wards of the city to ensure representativeness. Adults residing in the city since at least the past 3 months and who agreed to provide written informed consent for data and sample collection were included in the study. We excluded pregnant women, bed-ridden patients and those with recognisable cognitive impairment.

Minimum sample size required (*n*) was calculated for the finite population (*N* = 1 million) using Open Epi ver3.0 software using the following equation:

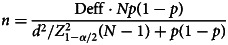


For the first round, we assumed a very low seroprevalence (*p*) of 1% as no data were available to date from other studies and with a relatively wide precision level (*d*) of 1%. For the subsequent rounds, we used the data from the previous survey to recalculate the sample size again (*p* = 2%±1% in round 2 and 6%±1.8% in round 3). A design effect of 1.6 and a non-response rate of 10% was added in each calculation. Thus, the necessary sample size for the first, second and third rounds was rounded off to 1000, 1500 and 1500, respectively.

### Survey procedure

**Fig. 1.**
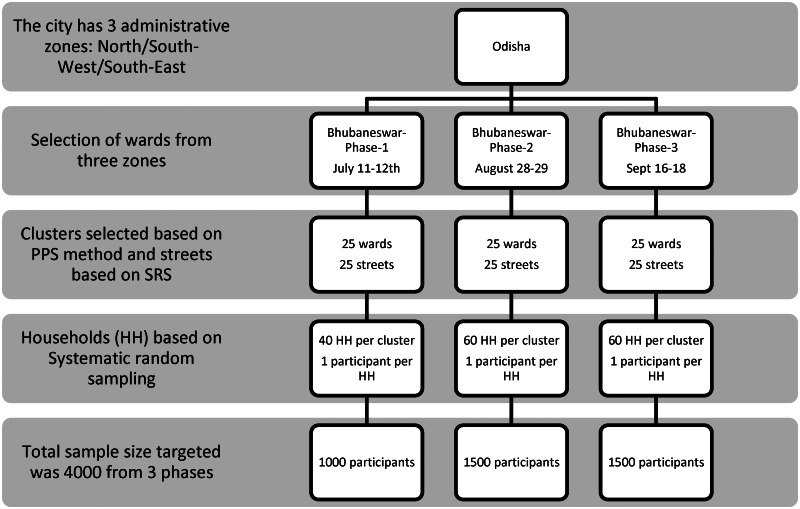
Sampling framework of multistage sampling for Bhubaneswar serosurvey.

Multi-stage random sampling was used for recruiting participants. The municipal wards were treated as clusters and 25 wards were selected randomly based on a probability proportional to size. Residential street names in the ward were listed and the street from where the sampling began (as well as the direction of sampling) in each cluster was selected by a computerised simple random method. Households in the street were selected using systematic random sampling and one eligible individual was selected from each household using an age-ordered matrix. Locked houses and/or non-response were recorded and the sampling frame was shifted to the immediate adjacent house in these cases. The details of the sampling framework are given in Figure 1.

### Data collection

Data on socio-demographic variables, exposure history to a confirmed (and/or suspected) case, symptom profile in the last 30 days, geographical location, travel and testing history were collected in a structured tool by trained field investigators who conducted participant interviews by door-to-door visits. An Open Data Kit-based electronic data capture tool (https://getodk.org/) was used for this purpose.

### Sample collection

Following all aseptic precautions, 3–4 ml blood samples were collected in the field by trained phlebotomists by venepuncture and transferred to vacutainers. These were transported maintaining a cold chain (2–8 °C) to the serology laboratory at Indian Council of Medical Research-Regional Medical Research Centre in Bhubaneswar (ICMR-RMRC) for analysis. Additionally, secondary data on the daily number of antigen tests carried out, the number of positives and deaths due to COVID-19 were obtained for the past 3 months from government sources directly.

### Laboratory procedure

Serum samples were subjected to detection in Roche Cobas e411 for the presence of total antibodies against COVID-19 using the electro-chemiluminescence immunoassay-based technique which is based on the principle of double-antigen sandwich assay and provides the result in 18 min. Elecsys^®^ Anti-SARS-CoV-2 is an immunoassay for the *in vitro* qualitative detection of antibodies (including immunoglobulin (Ig)G) to SARS-CoV-2 in human serum and plasma. The assay uses a recombinant protein representing the nucleocapsid (N) antigen for the determination of antibodies against SARS-CoV-2. The test is intended as an aid in the determination of the immune reaction to SARS-CoV-2.

Testing procedures were followed as per the manufacturer's instructions. Patient's samples (20 μl) were incubated with a mix of biotinylated and ruthenylated nucleocapsid (N) antigens. Double-antigen sandwich (DAGS) immune complexes are formed in the presence of the corresponding antibodies. After the addition of streptavidin-coated microparticles, the DAGS complexes bound to the solid phase via the interaction of biotin and streptavidin. After that, the reagent mixture was transferred to the measuring cell, where the microparticles were magnetically captured onto the surface of the electrode. Unbound substances were subsequently removed. Electrochemiluminescence was then induced by applying a voltage and measured with a photomultiplier. The signal yield increased with the antibody titre. The value was expressed in cut-off index (CoI) and a value of <1.0 was considered non-reactive and CoI ≥1.0 was reactive.

### Data analysis

The seroprevalence of SARS-CoV-2 infection was estimated as a proportion along with 95% confidence intervals (CIs) and its distribution assessed across rounds and demographic parameters. Gender weights were added to prevalence estimates to account for a higher non-response rate in females. The infection-to-case ratio and the IFR were calculated using the reported cases and death data obtained from government sources. Temporal comparisons of the community seroprevalence estimates with the detected number of cumulative cases, active cases, recoveries and deaths were done. Heat maps with varying seroprevalence were built for each of the city's wards. Statistical analyses were performed using R (ver. 4.0.2) software packages and GIS analysis was performed using QGIS (ver. 3.10).

#### Ethical consideration

Ethical approval for the protocol was obtained from the Institutional Human Ethics Committee at ICMR-RMRC Bhubaneswar and the State Health and Research Ethics Committee, Odisha. Written informed consent for participation was obtained and a participant information sheet was provided to each household. All methods were performed following ICMR-National ethical guidelines for biomedical research involving human participants. The findings of the study were shared with the relevant local communities by communications sent to the urban local administrative body's representatives and media releases in the local press.

Interviews were conducted ensuring privacy. All data were stored securely under the investigator's responsibility, with a focus on ensuring the confidentiality of study participants. The final report and publications are based on aggregate data without any identifying information. A database with electronic tracking, password-restricted access and audit trails, with time and date stamps on data entry and edits, was used for quality control. State Health Department and city administration authorities were actively engaged to ensure smooth operationalisation and to reduce any stigma among the residents. The study methods, analyses and reporting have been informed by the WHO Unity protocol and ICMR National Serosurvey protocol in India [8, 9].

## Results

**Fig. 2.**
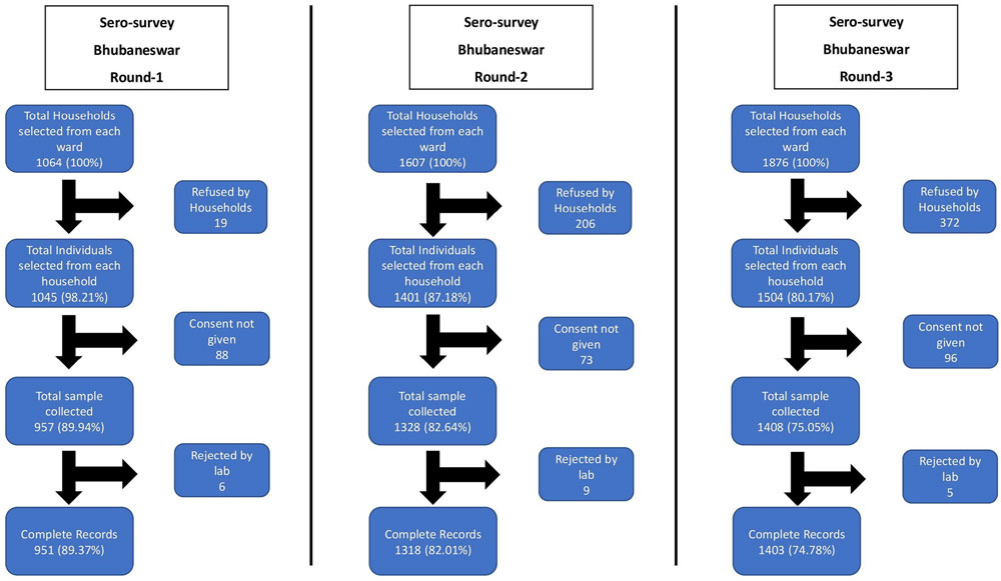
Study flow diagram of three rounds of Bhubaneswar serosurvey.

The repeated cross-sectional studies were conducted in three rounds in Bhubaneswar in which 3693 participants were enrolled in the study. We approached 4647 households from which 3693 participants provided blood samples, with a cumulative non-response rate of 18.33% in the three rounds. The non-response rate increased from the first phase (9.19%) to the third phase (24.94%). Of these, antibody test results were finally available for 3673 participants and these were included for the final analysis. The study flow diagram is provided in Figure 2.

The mean age of the study participants was 43.16 years (±13.93 years). The gender-weighted seroprevalence for the city in the first round was 1.55% (95% CI 0.84–2.58), the second round was 5.27% (95% CI 4.13–6.59) and in the third round was 49.04% (95% CI 46.39–51.68).

In the first round, the seroprevalence was found highest (1.68%) in the elderly population (age more than 60 years), whereas the seroprevalence for the second and third phases was highest (7.72% and 54.02% respectively) in the age group 30–39 years and the difference was significant across the age group. Seropositivity was higher in males (1.67%) in the first phase compared to females, but in the second and third phases, higher seroprevalence was found among females (5.37% and 50.12%). Overall, females reported a similar prevalence of 21.90% compared to males at 20.42%.

The demographic characteristics of the study population and the distribution of seroprevalence in each round are provided in Tables 1 and 2.

**Table 1.** Demographic characteristics of the study population

Characteristic	Round 1	Round 2	Round 3
	*n* = 951	*n* = 1319	*n* = 1403
Age group			
18–29 years	186 (19.55%)	276 (20.92%)	234 (16.67%)
30–39 years	179 (18.82%)	298 (22.59%)	311 (22.16%)
40–49 years	230 (24.18%)	311 (23.57%)	410 (29.22%)
50–59 years	177 (18.61%)	258 (19.56%)	283 (20.17%)
≥60 years	179 (18.82%)	176 (13.34%)	165 (11.76%)
Gender			
Male	718 (75.49%)	891 (67.55%)	982 (69.99%)
Female	233 (24.50%)	428 (32.44%)	421 (30.00%)
Occupation			
Public service	174 (18.29%)	217 (16.45%)	190 (13.54%)
Private service	198 (20.82%)	310 (23.50%)	347 (24.73%)
Self employed	178 (18.71%)	216 (16.37%)	343 (24.44%)
Manufacturing	2 (0.21%)	88 (6.67%)	55 (3.92%)
Homemaker	118 (12.40%)	252 (19.10%)	251 (17.89%)
Student	87 (9.14%)	73 (5.53%)	77 (5.48%)
Unemployed	31 (3.25%)	101 (7.65%)	56 (3.99%)
Others/retired	163 (17.13%)	62 (4.70%)	84 (5.98%)
Household size			
≤2	220 (23.13%)	239 (18.11%)	177 (12.61%)
3–4	482 (50.68%)	674 (51.09%)	765 (54.52%)
5–6	175 (18.40%)	307 (23.27%)	309 (22.02%)
>6	74 (7.78%)	99 (7.50%)	152 (10.83%)
History of respiratory symptoms in last 30 days	23 (2.41%)	57 (4.32%)	132 (9.40%)
History of contact with COVID-19 case	0 (0%)	0 (0%)	24 (1.71%)
Ever tested for COVID-19 by rRT-PCR	35 (3.68%)	185 (14.02%)	497 (35.42%)

**Table 2.** Distribution of seroprevalence for each round

Characteristic	Round 1		Round 2		Round 3	
	*n*	Seroprevalence (95% CI)	*n*	Seroprevalence (95% CI)	*n*	Seroprevalence (95% CI)
Age group						
18–29 years	186	1.61 (0.33–4.64)	276	5.80 (3.35–9.24)	234	42.74 (36.31–49.34)
30–39 years	179	0.56 (0.01–3.07)	298	7.72 (4.96––11.36)	311	54.02 (48.30–59.65)
40–49 years	230	1.30 (0.27–3.76)	311	2.57 (1.12–5.01)	410	51.71 (46.75–56.63)
50–59 years	177	2.82 (0.92–6.47)	258	3.49 (1.61–6.52)	283	49.12 (43.15–55.10)
≥60 years	179	1.68 (0.35–4.82)	176	7.39 (3.99–12.30)	165	38.18 (30.73 46.05)
Gender						
Male	718	1.67 (0.87–2.90)	891	5.16 (3.80–6.83)	982	47.96 (44.79–51.14)
Female	233	1.29 (0.27–3.72)	428	5.37 (3.44–7.95)	421	50.12 (45.24–54.99)
Occupation						
Public service	174	2.30 (0.63–5.78)	217	6.45 (3.57–10.59)	190	48.42 (41.12–55.76)
Private service	198	1.01 (0.12–3.60)	310	2.58 (1.12–5.02)	347	51.01 (45.61–56.38)
Self employed	178	2.25 (0.62–5.66)	216	5.09 (2.57–8.93)	343	45.77 (40.41–51.20)
Manufacturing	2	0.00 (0–0.84)	88	11.36 (5.59–19.91)	55	52.73 (38.80–66.34)
Homemaker	118	0.85 (0.02–4.63)	252	3.97 (1.92–7.18)	251	56.97 (50.59–63.18)
Student	87	1.15 (0.02–6.24)	73	4.11 (0.86–11.54)	77	41.56 (30.42–53.35)
Unemployed	31	0.00 (0–11.21)	101	7.92 (3.48–15.01)	56	42.86 (29.71–56.78)
Others/retired	163	1.84 (0.38–5.28)	62	8.06 (2.67–17.83)	84	33.33 (23.41–44.46)
Household size						
≤2	220	1.36 (0.28–3.93)	239	4.18 (2.02–7.56)	177	42.37 (34.99–50.01)
3–4	482	1.66 (0.72–3.24)	674	4.75 (3.27–6.64)	765	48.50 (44.90–52.10)
5–6	175	1.14 (0.14–4.07)	307	5.86 (3.51–9.11)	309	51.13 (45.40–56.83)
>6	74	2.70 (0.33–9.42)	99	9.09 (4.24–16.56)	152	51.32 (43.08–59.49)
Total	951	1.58 (0.88–2.58)	1319	5.23 (4.09–6.57)	1403	48.61 (45.96–51.26)

Seroprevalence was significantly higher among individuals with larger household sizes across all three rounds. Irrespective of the age group, the seroprevalence showed an increasing trend over the three time periods, with the highest seropositivity rates among individuals sampled between 16th and 18th September, 2020.

**Fig. 3.**
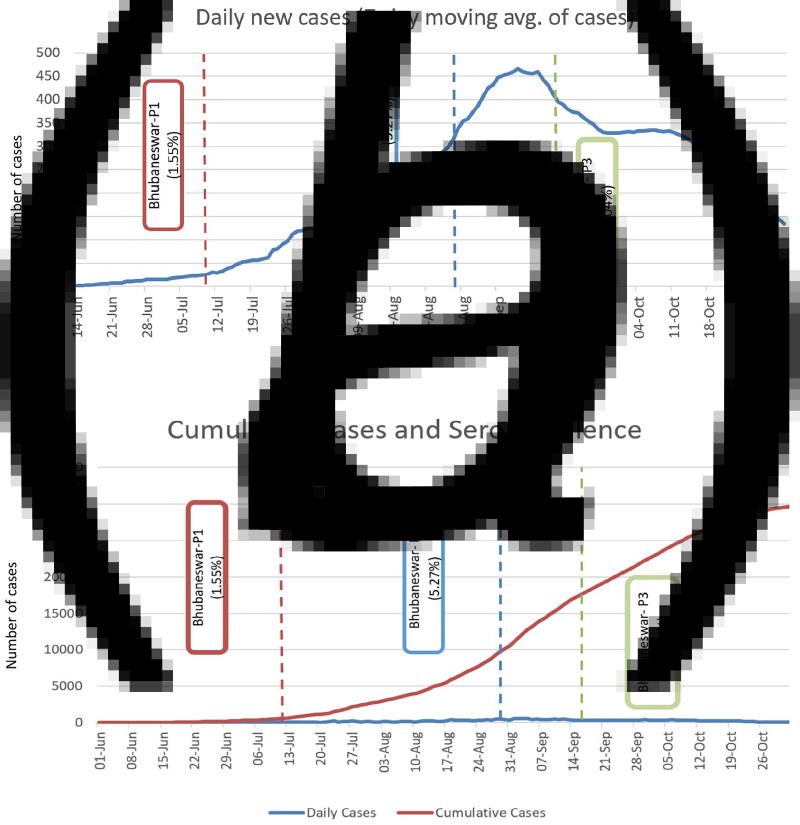
(a) Seven days moving average of new cases detected in three phases and point of seroprevalence. (b) Cumulative cases of COVID-19 and estimated sero-prevalence of Bhubaneswar.

The real-time reverse transcription polymerase chain reaction (rRT-PCR) testing rates among the population were increased with each round, culminating in a high proportion testing coverage of 35.5% by the third round. Mean duration from testing COVID-19 positive by rRT-PCTR to blood sample collection was 36.14 days (±18.27). Interestingly, by the third round, whereas 93.93% of those who had previously been tested positive by rRT-PCR had seroconversion at the time of sample collection, 46.57% of those who had been tested negative also showed seroconversion. The proportion of symptomatic individuals showed a steady increase from 2.41% in round 1 to 4.32% in round 2 and 9.40% in round 3. The seroconversion was also significantly more in symptomatic individuals across all three rounds as described in Table 3. Among the reported symptoms, the most common were fever (63.20%), followed by cough (51.88%) and myalgia (37.73%). The cumulative number of COVID-19 cases detected until 18th September, 2020, was 27 584 in Bhubaneswar. The association between time trends of the daily progression of new cases and cumulative cases and the time point of seroprevalence estimates is given in Figure 3a and b.

**Table 3.** Testing status and symptom profile of the study participants

Testing/symptom profile	Round 1		Round 2		Round 3	
	*N*	Seroprevalence (95% CI)	*N*	Seroprevalence (95% CI)	*N*	Seroprevalence (95% CI)
Tested by rRT-PCR						
Yes	35	2.85 (0.07–14.91)	185	7.02 (3.79–11.71)	497	59.15 54.68–63.51)
No	916	1.52 (0.83–2.55)	1134	4.93 (3.75–6.36)	906	42.82 (39.57–46.12)
Positive by rRT-PCR previously						
Yes	0	–	3	66.66 (9.42–99.15)	132	93.93 (88.40–97.34)
No	35	2.85 (0.07–14.91)	182	6.04 (3.05–10.55)	365	46.57 (41.36–51.83)
Symptoms of self (last 30 days)						
Yes	23	4.34 (0.11–21.94)	57	10.52 (3.96–21.51)	132	70.45 (61.89–78.07)
No	928	1.50 (0.82–2.51)	1262	4.99 (3.85–6.34)	1271	46.05 (43.29–48.82)

**Fig. 4.**
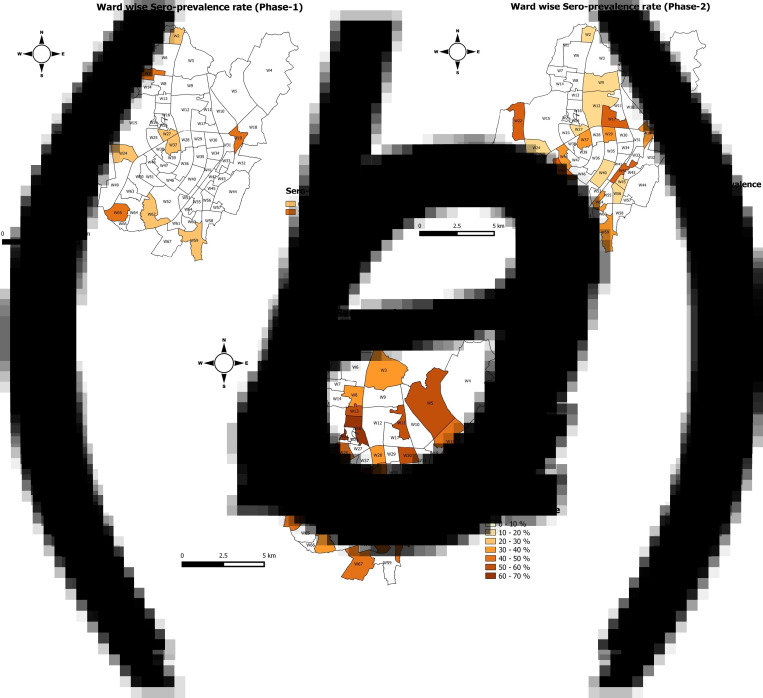
Wardwise seroprevalence of Bhubaneswar round 1, Bhubaneswar round 2 and Bhubaneswar round 3.

The infection-to-case ratio on the date of first round was 27.05, second round was 5.62 and third round was 17.91. The heat maps for the geographical distribution of the seroprevalence across the three cities are given in Figure 4a–c.

## Discussion

The first reported case of COVID-19 in the state of Odisha was detected in Bhubaneswar on 16th March 2020 [10]. We carried out a repeated cross-sectional seroepidemiological evaluation for SARS-CoV-2 infection in 25 wards of the city at three different time points between July and September, 2020. Analysis of seroprevalence of anti-SARS-CoV-2 antibodies showed a trend of increasing seropositivity in the sampled population at the three time points from July 2020 to September 2020. The findings from these serosurvey rounds were meticulously considered by the State Government and Bhubaneswar Municipality Corporation for framing the unlock strategies to come out from the enforced mass movement restrictions. The highest seropositivity was during the 16th to 18th September 2020 sampling period with positivity of 49.04%. The earliest national level serosurvey carried out in India during May to June, 2020, during the phase of ongoing national level lockdowns had reported low overall seropositivity rates in most districts which had been included [3]. Subsequent surveys from different parts of India have reported higher rates of seropositivity which have corresponded to the phased reopenings in different parts. In a study performed during July and August, 2020, in five administrative sub-wards of Pune city in Maharashtra, the state with the highest number of COVID-19 cases until date, seropositivity rates were substantially high (51.3%, 95% CI 39.9–62.4) but showed wide variation among the included localities [11]. In a serosurvey set in Mumbai including slums and non-slum settings in July, 2020, 54.1% of samples from slums compared to 16.1% of samples from non-slum areas were found to be positive for anti-SARS-CoV-2 antibodies [12]

In the repeated cross-sectional serosurveys in Bhubaneswar, the highest seropositivity of 49.04% during 16th to 18th September, 2020, was preceded by the highest 7-day average of the number of new COVID-19 cases as detected by rRT-PCR or antigen testing in the city. The 7-day average number of new COVID-19 cases has subsequently shown a declining trend, which might be indicative of some extent of approaching community level immunity at least in the short term. Similar trends have also been reported in serosurveys from slum settings in Mumbai [12]. However, it is emphasised that the interpretation of such trends must be dealt with caution, given the heterogeneity of seroprevalence and seropositivity at the level of different wards and the still evolving understanding of the antibody kinetics and antibody response longevity against SARS-CoV-2. Although the overall phase three seropositivity of 49.04% might be considered an encouraging trend for approaching community level immunity, it falls significantly short of 67%, which has been suggested as the threshold for herd immunity for SARS-CoV-2 as per published estimates [13].

The estimated infection-to-case ratios during the three testing phases were 27.05 (July, 2020), 5.62 (August, 2020) and 17.91 (September, 2020). The lowest infection-to-case ratio in August corresponded to a steep increase in the 7-day moving average number of new cases. Infection-to-case ratio information might be of help to local, state and national authorities while prioritising regions where case detection might need to be ramped up [14].

### Seroprevalence: gender

Although the overall seroprevalence was comparable between the two genders, in our overall analysis, we found a higher seroprevalence among women in round 2 and round 3 of the serosurvey. Similar to our study, a recent evaluation of seroprevalence of SARS-CoV-2 antibodies among a population in Delhi revealed the highest antibody positivity among women ≥50 years of age [15]. A serosurvey in the slum and non-slum settings in Mumbai carried out in July, 2020, also reported significantly higher unadjusted positive proportions among women compared to men in both slum and non-slum settings [12]. However, a recent preprint of a systematic review and meta-analysis of global trends on SARS-CoV-2 seroprevalence based on 56 studies has suggested a lack of gender-based differences in seroprevalence [14].

### Seroprevalence: age groups

Although the highest rates of seropositivity were found among the elderly (>60 years age) and 18–29 age group during the first round of the serosurvey in July, 2020, in the latter phases in August and September, 2020, the highest seropositivity rates were observed among adults aged 30–39 years. Data from serosurveys carried out in Mumbai, India, suggest a higher positivity among the elderly population compared to adults (25–39 years) in slum settings, whereas significantly lower positivity was seen in elderly residing in non-slum settings [12]. This might be related to the overcrowded conditions in slums and low socio-economic settings as well as better awareness regarding SARS-CoV-2 transmission and care for the elderly in more well-off settings.

### Seroprevalence and rRT-PCR testing and symptoms

In this study, IgG antibodies against SARS-CoV-2 were detected in more than 90% of all individuals who participated in the serosurvey and had previously been diagnosed as a case of COVID-19. However, serosurveys in Delhi have reported much lower seropositivity among serosurvey participants with a history of COVID-19 diagnosed either by rRT-PCR or antigen-based tests [15]. Testing coverage with consistent kit performance has increased from first serosurvey to third serosurvey.

### Importance of future serosurveys

Although the three phases of serosurveys carried out in the city of Bhubaneswar, India, show an increasing trend of seropositivity, it is important to conduct such surveys in the coming months to inform regarding the population-level dynamics of anti-SARS-CoV-2 antibodies. Repeated serosurveys from Delhi have shown a declining trend from 28.36% in August, 2020 to 25.1% in September, 2020 in SARS-CoV-2 antibody prevalence [15, 16]. Similar trends have also been reported in serosurveys from the UK [17]. This might be indicative of the possibility of the waning of population-level immunity to SARS-CoV-2. SARS-CoV-2 specific antibody levels have been reported to decline rapidly in individuals with mild COVID-19 disease [18]. Many different factors including disease severity, age and pre zexisting comorbidities are probably important determinants of antibody response longevity [19].

Our study had a number of shortcomings, the assays used for detecting antibodies were qualitative and semi-quantitative at best and hence the geometric mean titres of IgG antibodies in the different phases of testing could not be calculated. We confined the serosurveys to adults, and age groups <18 years were not represented due to which the age weighted prevalence was not calculated. It will be important to include these age groups in future sero-epidemiological studies on SARS-CoV-2. Moreover, since we included only single individuals from each household in the serosurveys, there is the potential of underestimating the seroprevalence as disease transmission is expected to be higher within households. Compared to the first and second round serosurveys where the non-response rates in the included communities were low, non-response in the third round of serosurvey was significantly higher at 24.14%.

When compared to high-income and western settings, the number of sero-epidemiological studies on SARS-CoV-2 performed and reported from low- and middle-income settings are few. Our study presents the sero-epidemiological status of the city of Bhubaneswar at three different time points, showing an increasing trend of seropositivity among the adult population which was sampled. The three rounds of serosurveys correspond to three phases of nationwide lockdown and the subsequent phased reopenings which were carried out across India as a response to the ongoing COVID-19 pandemic and provide valuable insights into the dynamics of SARS-CoV-2 infection and its spread during the different phases. The findings from our study may prove useful to model seroprevalence trends in other regions which have not been carried out. The risk factors identified in our serosurvey can be used to create models for projection of the progression of the pandemic in similar tier-2 cities across India. Although comparing seroprevalence rates between different regions, states and countries, it is important to understand the caveats associated with the variability of the numerous test platforms and methods used for antibody detection for SARS-CoV-2 as well as other regional and local factors which may affect the inferences. Moving forward, efforts should be made to use standardised methods in serosurveys to ensure the comparability of results in different settings and locations.

## Conclusion

Our study found a sudden increase of seroprevalence against COVID-19 in Bhubaneswar, which might be due to the unlocking of various parts of the city after initial lockdown. The first serosurvey was conducted during the lockdown period, whereas the second serosurvey was during the initial opening of the lockdown phase. The third serosurvey was conducted after complete unlocking of the city as per the guidelines by the Government of India. The study covers the three phases of the lockdown event and their seroprevalence during each phase. Prevalence was low during the first and second round of surveys, possibly due to travel restrictions during the lockdown and high adherence of the population to social distancing and wearing masks. Understanding the dynamics of COVID-19 transmission and the susceptibility to infection at the individual and community levels will provide local data on the critical parameters of the pandemic, which can be used by decision-makers to design and implement effective public health policies to mitigate the burden of COVID-19 in the state.

## Data Availability

The datasets used and/or analysed during the current study are available on request to the corresponding author.

## Supplementary material

For supplementary material accompanying this paper visit http://dx.doi.org/10.1017/S0950268821000972. click here to view supplementary material
