# Supplementary material for: Seroprevalence of SARS-CoV-2 in Bhubaneswar, India: findings from three rounds of community surveys
Source: Epidemiol Infect. 2021 Apr 27;149:e139. doi: 10.1017/S0950268821000972 (PMC8207548; doi:10.1017/S0950268821000972)
Supplement: Supplementary file 1 [file hygsup.zip › S0950268821000972sup001.docx]

Annexure-2

Gender distribution of decline of consent

| Round | Male | Female | Total |
| --- | --- | --- | --- |
| Serosurvey-1 | 28 | 60 | 88 |
| Serosurvey-2 | 13 | 60 | 73 |
| Serosurvey-3 | 11 | 85 | 96 |
|  |  |  |  |
