# Supplementary material for: Seroprevalence of SARS-CoV-2 in Bhubaneswar, India: findings from three rounds of community surveys
Source: Epidemiol Infect. 2021 Apr 27;149:e139. doi: 10.1017/S0950268821000972 (PMC8207548; doi:10.1017/S0950268821000972)
Supplement: Supplementary file 1 [file hygsup.zip › S0950268821000972sup002.docx]

**First Sero Survey**

| **Sl.No.** | **Wards selected** | **Households (Total)** | **Starting point Locality selected (SRS)** | **Households to be sampled/ cluster**  **(1 participant per HH)** |
| --- | --- | --- | --- | --- |
|  | 2 | 2708 | Patia Big Bazaar area | 40 |
|  | 4 | 3476 | Hanspal | 40 |
|  | 7 | 3420 | SailashreeVihar GA Plot area | 40 |
|  | 9 | 3328 | Aditya Care Hospital area | 40 |
|  | 12 | 3227 | Doordarshan Kendra | 40 |
|  | 14 | 3255 | Budhha Park area | 40 |
|  | 17 | 2786 | Postal Colony | 40 |
|  | 19 | 3517 | Basudev Nagar | 40 |
|  | 22 | 3207 | Durga Madhab area | 40 |
|  | 24 | 3331 | RBI Colony | 40 |
|  | 27 | 2958 | Nayapalli PS Area | 40 |
|  | 29 | 2796 | Irrigaiton Colony | 40 |
|  | 32 | 2518 | Bankers Colony | 40 |
|  | 35 | 3422 | Nalini Devi College | 40 |
|  | 37 | 2831 | Nayapalli Behera Sahi | 40 |
|  | 40 | 2808 | Jayadev Bhawan | 40 |
|  | 42 | 3438 | Gokula Jena Colony area | 40 |
|  | 45 | 2749 | Biswal Complex | 40 |
|  | 48 | 2767 | KrushiVihar | 40 |
|  | 51 | 2904 | PrasantiVihar | 40 |
|  | 54 | 2539 | Gyana Nagar | 40 |
|  | 56 | 2855 | BJB Nagar | 40 |
|  | 59 | 2855 | Sriram Nagar area | 40 |
|  | 62 | 2813 | LingarajVihar | 40 |
|  | 65 | 2823 | Dumduma PH-I | 40 |
| Total | 25 wards | 75331 HH | 25 | 1000 participants |

**Second Sero Survey**

| **Sl.No.** | **Wards selected** | **Households (Total)** | **Starting point Locality selected (SRS)** | **Households to be sampled/ cluster**  **(1 participant per HH)** |
| --- | --- | --- | --- | --- |
|  | 2 | 2708 | Munda Sahi | 60 |
|  | 4 | 3476 | Haridaspur | 60 |
|  | 7 | 3420 | Niladri Enclave Area | 60 |
|  | 9 | 3328 | Gadakan Village | 60 |
|  | 12 | 3227 | Samanta Vihar | 60 |
|  | 14 | 3255 | Lumbini Vihar | 60 |
|  | 17 | 2786 | Utkal University Area | 60 |
|  | 19 | 3517 | Hanuman Sahi | 60 |
|  | 22 | 3207 | Mahalaxmi Vihar, | 60 |
|  | 24 | 3331 | Jagannath Vihar Area | 60 |
|  | 27 | 2958 | IRC Village N-5 & N-6 | 60 |
|  | 29 | 2796 | GRIDCO Colony, | 60 |
|  | 32 | 2518 | Laxmisagar Brit Colony | 60 |
|  | 35 | 3422 | Exhibition Ground | 60 |
|  | 37 | 2831 | Trishna Apartment | 60 |
|  | 40 | 2808 | Kamala Neheru Womens College | 60 |
|  | 42 | 3438 | Budhhanagar | 60 |
|  | 45 | 2749 | Post Office Lane | 60 |
|  | 48 | 2767 | Stewart School | 60 |
|  | 51 | 2904 | OUAT Office | 60 |
|  | 54 | 2539 | Mishra Sahi | 60 |
|  | 56 | 2855 | Mainisia Khala | 60 |
|  | 59 | 2855 | Haripur Patna | 60 |
|  | 62 | 2813 | Ananta Vihar | 60 |
|  | 65 | 2823 | Kanungo Diabetes Hospital | 60 |
| Total | 25 wards | 75331 HH | 25 | 1500 participants |

**Third Serosurvey**

| **Sl.No.** | **Wards selected** | **Households (Total)** | **Starting point Locality selected (SRS)** | **Households to be sampled/ cluster**  **(1 participant per HH)** |
| --- | --- | --- | --- | --- |
| 1 | 3 | 2708 | Shree Vihar | 60 |
| 2 | 5 | 3476 | Satya Vihar | 60 |
| 3 | 8 | 3420 | Prachi Enclave | 60 |
| 4 | 11 | 3328 | VSS Nagar Housing Board Area | 60 |
| 5 | 13 | 3227 | Rail Vihar | 60 |
| 6 | 16 | 3255 | Maitri Vihar | 60 |
| 7 | 18 | 2786 | Palasuni hata | 60 |
| 8 | 21 | 3517 | Jaydev Nagar | 60 |
| 9 | 25 | 3207 | N-3 | 60 |
| 10 | 28 | 3331 | Acharya Vihar New AG Colony | 60 |
| 11 | 30 | 2958 | Sahid Nagar Area | 60 |
| 12 | 33 | 2796 | Jaydurga nagar | 60 |
| 13 | 36 | 2518 | Unit-4 Girls High School | 60 |
| 14 | 39 | 3422 | Naypalli Nua Sahi | 60 |
| 15 | 41 | 2831 | Nilachal Hospital Area | 60 |
| 16 | 44 | 2808 | Badagada Village | 60 |
| 17 | 46 | 3438 | Surya Nagar | 60 |
| 18 | 49 | 2749 | Ruchika Market Area | 60 |
| 19 | 52 | 2767 | Pallashpalli Area | 60 |
| 20 | 55 | 2904 | Goutam Nagar | 60 |
| 21 | 58 | 2539 | Rajarani Colony | 60 |
| 22 | 60 | 2855 | Gouri Nagar | 60 |
| 23 | 63 | 2855 | Jagamohan Nagar | 60 |
| 24 | 64 | 2813 | Pabani Complex | 60 |
| 25 | 67 | 2823 | Kapil Prasad BDA colony | 60 |
| Total | 25 wards | 75331 HH | 25 | 1500 participants |
